# A micromorphological/microbiological pilot study assessing three methods for the maintenance of the implant patient

**DOI:** 10.1002/cre2.345

**Published:** 2020-11-19

**Authors:** Elisabetta Polizzi, Bianca D'orto, Simone Tomasi, Giulia Tetè

**Affiliations:** ^1^ Department of Dentistry, IRCCS San Raffaele Hospital Vita Salute University Milan Italy; ^2^ Freelance Dental Hygienist Milan Italy

**Keywords:** implants surface, maintenance therapy, osteoblasts, peri‐implantitis, piezoelectric ultrasonic

## Abstract

**Objective:**

The aim of this study was to evaluate and compare the effectiveness of the ultrasonic piezoelectric inserts of EMS Steel Tip A, EMS Peek, and IS‐TiP‐STS‐3E^©^ in reducing peri‐implant bacterial load without compromising the surface of implants during professional oral hygiene in the follow‐up.

**Materials and methods:**

Thirteen implants were examined (Winsix, Biosafin, Ancona, Italy). The implants were divided into five groups and analyzed with a SEM microscope and microbiological analysis to evaluate the possible modification of structure and the bacterial load reduction.

**Results:**

The control and A, B, and C test groups were initially contaminated in vitro with Streptococcus mutans. Subsequently, the A, B, and C test groups were treated by an only expert operator in standard conditions. Test groups A, B, and C were inoculated for 3 hr and, furthermore, microbiologically analyzed.

**Conclusion:**

The gold standard of an implant maintenance is a significant reduction of the bacterial load without becoming aggressive. According to our results, despite the limitations of the study, the authors recommend the least aggressive IS‐TiP‐STS‐3E^©^, but combined with an antimicrobial agent to reduce the bacterial load, because the IS‐TiP‐STS‐3E^©^ did not show appreciable results versus the EMS Peek in reducing the bacterial load.

## 1 INTRODUCTION

Osseointegrated dental implants, supporting fixed prostheses, are an effective therapeutic alternative to replace missing teeth (Cattoni et al., 2020).

When bone level is too reduced compared to traditional placement of straight implants, different procedures are proposed as an alternative, in order to obtain fixed rehabilitation: bone grafting (Salvato & Agliardi, 2007; Vinci, Tetè, Raimondi Lucchetti, Capparè, & Gherlone, 2019), crest augmentation (Crespi, Capparè, Polizzi, & Gherlone, 2014; McAllister & Haghighat, 2007), sinus floor elevation techniques (lateral approach or osteotome‐mediated technique) (Lundgren et al., 2017), short implants,(Kim, Ku, Kim, Yun, & Kim, 2018) and tilted implants (Capparé et al., 2019; Krekmanov, Kahn, Rangert, & Lindström, 2000).

Despite the surgical technique, maintenance of professional hygiene is fundamental for the success of implant‐prosthetic rehabilitation as peri‐implantitis can be considered as a direct consequence of poor oral hygiene (Lindhe & Meyle, 2008).

The accumulation of bacterial plaque around the implants associated with other causes, such as absence of keratinized mucosa, systemic pathologies, smoking habits, and occlusal overload, could cause implant loss (Klinge, 2012; Levin, 2013).

During the hygienic maintenance therapy, several instruments for debridement were proposed: curettes, air polishing, and ultrasound devices.

The association of chemical agents and local antimicrobials with mechanical instrumentation has shown benefits in implant maintenance therapy (Calderini, Pantaleo, Rossi, Gazzolo, & Polizzi, 2013).

The aim of this study was to evaluate and compare the effectiveness of the EMS Steel Tip A, EMS Peek, and IS‐TiP‐STS‐3E^©^ ultrasonic piezoelectric inserts in reducing the peri‐implant bacterial load without compromising the implant surface during professional oral hygiene in the follow‐up. According to several studies, mechanical tools during professional hygiene are recommended; however, they can damage the implant surface (Cha et al., 2019; Louropoulou, Slot, & Van der Weijden, 2012).

Furthermore, ultrasonic instruments, compared to manual procedures, can be considered more effective (Vyas et al., 2019; Vyas, Grewal, Kuehne, Sammons, & Walmsley, 2020; Walmsley, Walsh, & Et, 1990).

## 2 MATERIALS AND METHODS

Thirteen sand‐blasted and acidified implants, having a diameter of 4.5 mm and a length of 15 mm, were examined by gamma ray sterilization, (Winsix, Biosafin, Ancona, Italy). The implants were divided into five groups consisting of:

- SEM control: one untreated implant.
- Microbiological control: three inoculated and untreated implants.
- Test group A: three contaminated implants and treated with EMS steel tip A.
- Test group B: three contaminated implants and treated with EMS Peek tip.
- Test group C: three contaminated implants and treated with IS‐TiP‐STS‐3E^©^ tip.

An in vitro model was recreated, and the microbiological analysis was conducted by experts at our university microbiology laboratory; while for the morphological analyzes, an expert conducted the preparations and analyzes in a double‐blind.

### 2.1 Bacterial contamination protocol

Bacterial colonies of Streptococcus mutans were initially pre‐inoculated from a frozen stock and allowed to grow in 1 ml of Todd Hewitt Broth culture medium (Manufacturer: Becton Dickinson Sparks, MD) overnight.

The following day, the bacterial solution was diluted in order to obtain an O.D (optical density) of 480 nm = 0.1 CFU (Colony forming units, i.e., the bacterial quantity). The optimal wavelength for Streptococcus mutans colonies has been validated with previous experiments (Lundgren et al., 2017).

Standard curves for OD determinations were generated and compared with direct bacteria count by Acridine orange and subsequent visualization below epifluorescence microscope.

The two bacterial counts were consistent with each other; the OD measurement was thus used for subsequent determinations. The bacteria solution, OD 480 Nm = 0.1 CFU, was employed in order to contaminate both microbiological control implants and A, B, and C test groups' implants.

The inoculation was carried out under stirring, in the bacterial solution, for 10 hr. Subsequently, the A, B, and C test groups were subjected to the instrumentation treatment as per protocol. Following the instrumental treatment, the implants were inoculated for further 3 hr, under stirring, in a sterile medium. The soil solutions obtained were used for the measurement of the OD. Before being sent to the SEM scanning procedures, the implants were subjected to a bacterial fixing treatment on the surface by immersing 16% Glutaraldehyde for 30 min and then washed with distilled water.

### 2.2 Instrumentation protocol

Clinical procedures of scaling near the area of the peri‐implant sulcus were reproduced in vitro. The instrumentation steps were carried out in a horizontal and oblique direction in relation to the long axis of the implant. To define and encode an average instrumentation time for the samples under examination, the literature was examined and a maximum time of mechanical instrumentation of 4–6 min was applied (Decker, 2001).

For the study in question, simulating a supragingival instrumentation or mostly in the gingival sulcus, a time of 60 min was agreed for the instrumentation of each sample. As regards the working power of the Piezon EMS ultrasound device, a minimum power of 20,000 cycles per second has been calibrated.

All the movements of the instrumentation around the systems were carried out by the same expert operator in standard conditions.

Group A was assigned a mechanical instrumentation with Piezon ultrasonic handpiece (EMS) with steel tip “A” (EMS); the three samples making up the group were instrumented with the coded time of 60 min, with horizontal and oblique movements on the circumference of the implant.

Group B was assigned a mechanical instrumentation with Piezon ultrasonic handpiece (EMS) with Peek tip (EMS); the three samples making up the group were instrumented with the coded time of 60 min, with horizontal and oblique movements on the circumference of the implant.

Group C was assigned a mechanical instrumentation with Piezon ultrasound handpiece (EMS) with IS TiP STS‐3E © tip; the three samples making up the group were instrumented with the coded time of 60 min, with horizontal and oblique movements on the implant circumference.

After instrumentation on the A, B, and C test groups, the microbiological analysis was carried out, and subsequently they were sent to the ESEMIR Sas laboratory (Di Battaini Paolo & C); where they were initially subjected to 1 metallization cycle with gold (the instrument used is an Agar Auto Sputter Coater of the company AGAR SCIENTIFIC LTD), in order to make them electron conductive and, therefore, analyzed with the scanning electron microscope (SEM).

SEM observations were performed with a Cambridge Stereoscan 120 instrument digitized with the Adda II system and equipped with electronic energy dispersion microanalysis (EDS). The main parameters of the analysis—electron acceleration potential (=EHT), Working distance (=WD), and magnification (=MAG)—are shown on the black strip at the bottom of each scan. Three different magnification images were taken, both for the SEM control and for the A, B, and C test groups. Magnifications were made at ×100, ×500, and ×1,000 on the A–B–C test groups, an image for each sample belonging to the group. The individual scans were compared for the same magnification with the SEM control.

A, B, and C test groups were compared with the control group in order to evaluate the possible damages induced by mechanical instrumentation with different treatments, so as to identify possible modifications made to the treated implant structure. After a ×500 magnification, considered the most significant for image quality, the highest definition images of different instruments were selected: EMS steel tip A, EMS Peek, IS‐TiP‐STS‐3E^©^, and thus compared with each other and with the SEM control with the same magnification.

## 3 RESULTS

### 3.1 Microbiological results

From the analyzes carried out after the instrumentation on the A, B, and C test groups with respect to the microbiological control, it was found that:

**Group A**: there was no reduction of the bacterial count, but an increase of it in the following 3 hours of inoculation with a standard deviation of 3%.

**Group B**: a highly significant reduction in bacterial count occurred with a standard deviation of 8%.

**Group C**: a significant reduction in bacterial count occurred with a standard deviation of 4%.

**FIGURE 1:**
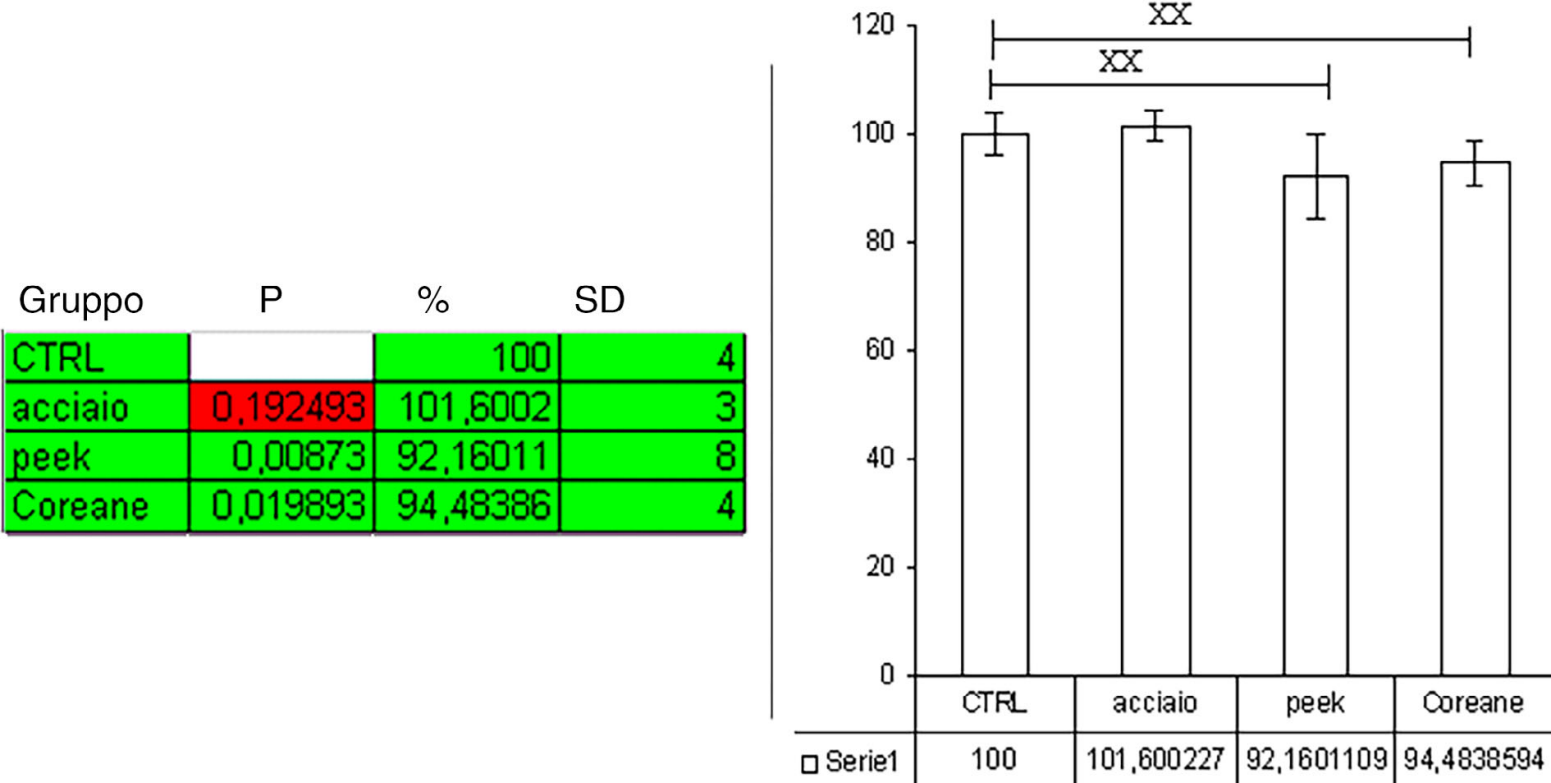
The graph shows for each individual insert the degree of effectiveness in removing the amount of surface bacteria. It can be seen that the insert with the highest bacterial removal capacity is the steel one, in second place the Korean tips, and lastly the peek tip

It is recalled, looking at the graph (Figure 1), that for P to have statistical significance, it must be less than 0.01. It is, therefore, deduced that the implants of groups B and C have reached a high statistical significance of bacterial reduction, *p* < .01.

### 3.2 Morphological results

Test group (A) vs SEM control at ×500.

**FIGURE 2:**
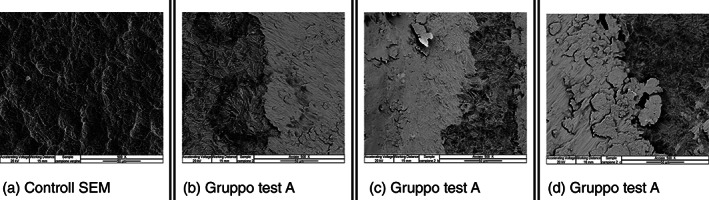
In the ×500 scans instrumented with EMS steel insert (b–d) we can see, with respect to the virgin control (a), areas of scratching and surface smoothing with the presence of titanium “flakes” presumably caused by the instrumentation made with EMS steel tip. Gruppo test (A) versus Controllo SEM a ×500

In the 500x scans, in addition to the scratching and surface smoothing areas, titanium “flakes,” presumably caused by the instrumentation performed with an EMS steel tip, are appreciated. (Figure 2a–d).

Test group (B) Vs SEM control at ×500.

**FIGURE 3:**
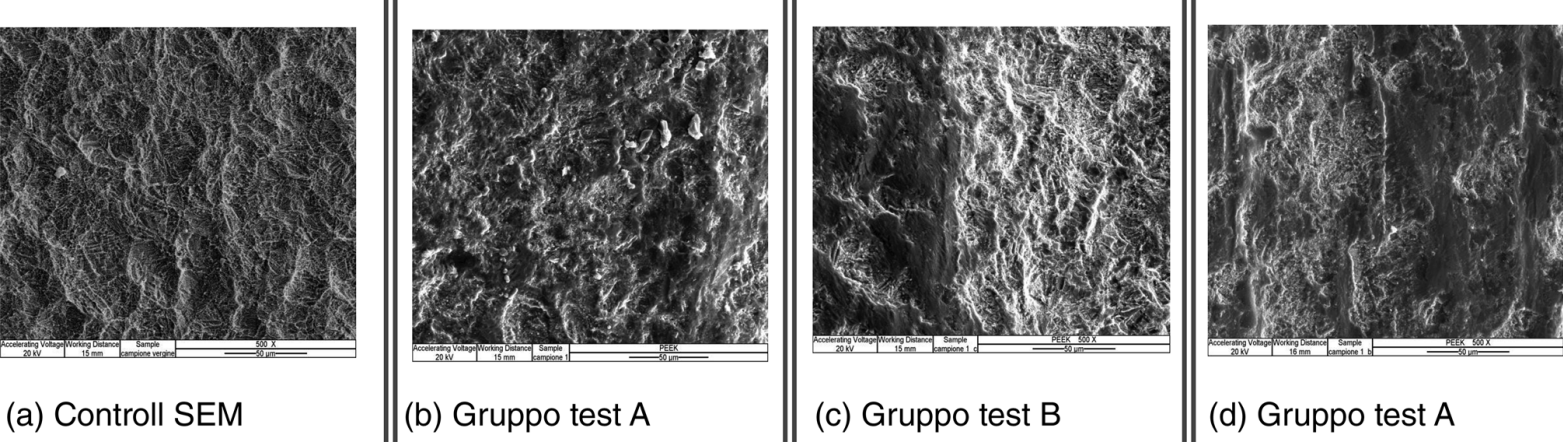
In the ×500 scans instrumented with a peek insert (b–d), compared to the virgin control (a), the areas of marked sanding of the surface with a vertical and horizontal trend, due to the action of the Peek tip mounted on the ultrasonic handpiece, remain more visible between the normal implant texture of the implant. Gruppo test (B) versus Controllo SEM a ×500

In the 500x scans, the marked sanding areas of the surface remain more visible between the normal weaving of the implant with a vertical and horizontal trend, due to the action of the Peek tip mounted on the ultrasound handpiece. (Figure 3a–d).

Test group (C) Vs SEM control at ×500.

**FIGURE 4:**
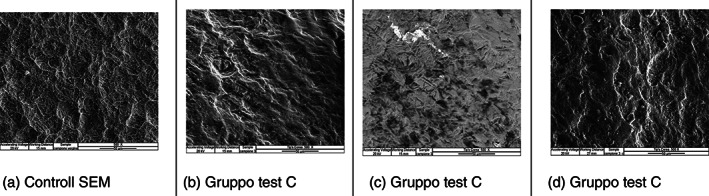
In the ×500 scans, instrumented by means of the IS TiP insert (b–d) with respect to the control (a) it can be seen that the insert has not considerably modified the implant surface, a slight smoothing of the surface is observed, due to the action of the tip mounted on the ultrasound handpiece. Also, at this magnification the partial preservation of the surface morphology of the implant origin can be observed. Scan No. 12 showed the presence of a clear deposit; when analyzed by metallography it was found to be the alloy of which the IS‐TiP‐STS‐3E insert is composed (Figure 6). Gruppo test (C) versus Controllo SEM a ×500

In the 500x scans, it can be seen that the insert has not significantly changed the implant surface, there is a slight smoothing of the surface, due to the action of the tip mounted on the ultrasound handpiece. Even at this magnification, the partial conservation of the surface morphology of the origin of the implant can be observed. (Figure 4a–d).

In the scan no 41, the presence of a clear deposit was verified; analyzed by metallography, it was found to be the alloy of which the IS‐TiP‐STS‐3E^©^ insert is composed (Figure 2d) in comparison at ×500 of the test groups A, B, and C Vs SEM control.

**FIGURE 5:**
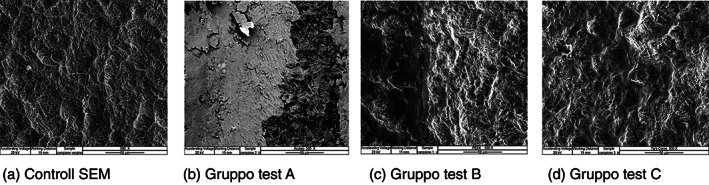
Most significant images of the three types of inserts used for mechanical instrumentation (b–d) compared with the control (a). Comparazione a ×500 dei Gruppo test A‐B‐C versus Controllo SEM

The images of all the instruments (Steel A EMS, Peek EMS, IS‐TiP‐STS‐3E^©^) were compared at a magnification of ×500, which is considered the most significant image quality. It was pointed out that the IS‐TiP‐STS‐3E^©^ tip (Figure 5a–d) was the least aggressive compared to the Peek and Steel inserts (A). The Peek tip (Figure 5b) was, in turn, less aggressive than the steel insert. The steel insert was, therefore, the most aggressive (Figure 5c).

## 4 DISCUSSION

Osseointegrated dental implants supporting fixed prostheses could effectively be considered the gold standard in the rehabilitation of patients with partial or total edentulism (Cattoni et al., 2020; Spitznagel, Horvath, & Gierthmuehlen, 2017).

Implant survival depends on the effectiveness of maintenance therapy and the selection of different instruments, such as curettes, ultrasonic scalers, air‐powder abrasive systems, lasers, chemicals, and local antimicrobials (Brookshire, Nagy, Dhuru, Ziebert, & Chada, 1997; Duarte, Reis, de Freitas, & Ota‐Tsuzuki, 2009; Garcia Canas, Khouly, Sanz, & Loomer, 2015).

After mechanical instrumentation, any damage to the surface of the implants can be recorded (Cha et al., 2019; Louropoulou et al., 2012).

As reported by several studies, the alteration of the implant surface negatively affects the proliferation and response of osteoblasts, so their attachment may be easier on surfaces with rougher microtopography (Bordji et al., 1996; Lincks et al., 1998).

The use of ultrasonic scalers may cause various changes on the implant surfaces (Harrel, Wilson Jr, Pandya, & Diekwisch, 2019; Ramaglia, di Lauro, Morgese, & Squillace, 2006).

Therefore, only professional oral hygiene is not sufficient for implant maintenance, and good oral health needs to be improved with home hygiene (Tecco et al., 2018).

The aim of this study was to evaluate and compare the effectiveness of the EMS Steel Tip A, EMS Peek, and IS‐TiP‐STS‐3E^©^ ultrasonic piezoelectric inserts in reducing the peri‐implant bacterial load without compromising the implant surface during professional oral hygiene in the follow‐up.

The data obtained in our in vitro study are similar to the evidence‐based and in vivo studies of many Authors (Matarasso et al., 1996; Rapley, Swan, Hallmon, & Mills, 1990).

The SEM images had ×100, ×500, and ×1,000 magnifications, and showed how all test groups (A, B, and C) analyzed reported visible damage on the implant surface.

Our results showed that the EMS A tip was too aggressive, the EMS Peek tip was mildly aggressive, and IS TiP STS‐3E^©^ caused minor surface damage to be considered almost irrelevant. However, after the metallography analysis, in scan no 42 at ×500 magnification, an evident deposit of IS‐TiP‐STS‐3E^©^ was found, which is considered irrelevant given its composition (gold alloy) and the remote randomness of identification (Figure 5d).

Our results are in accordance with scientific evidence; several studies, in fact, underline that mechanical instruments leave traces on the surface of the implant with removal of substance (Mengel, Buns, Mengel, & Flores‐de‐Jacoby, 1998).

However, some authors show that a nonmetallic ultrasonic tip is less aggressive, and results similar to those of the control groups are obtained (Bailey, Gardner, Day, & Kovanda, 1998).

**FIGURE 6:**
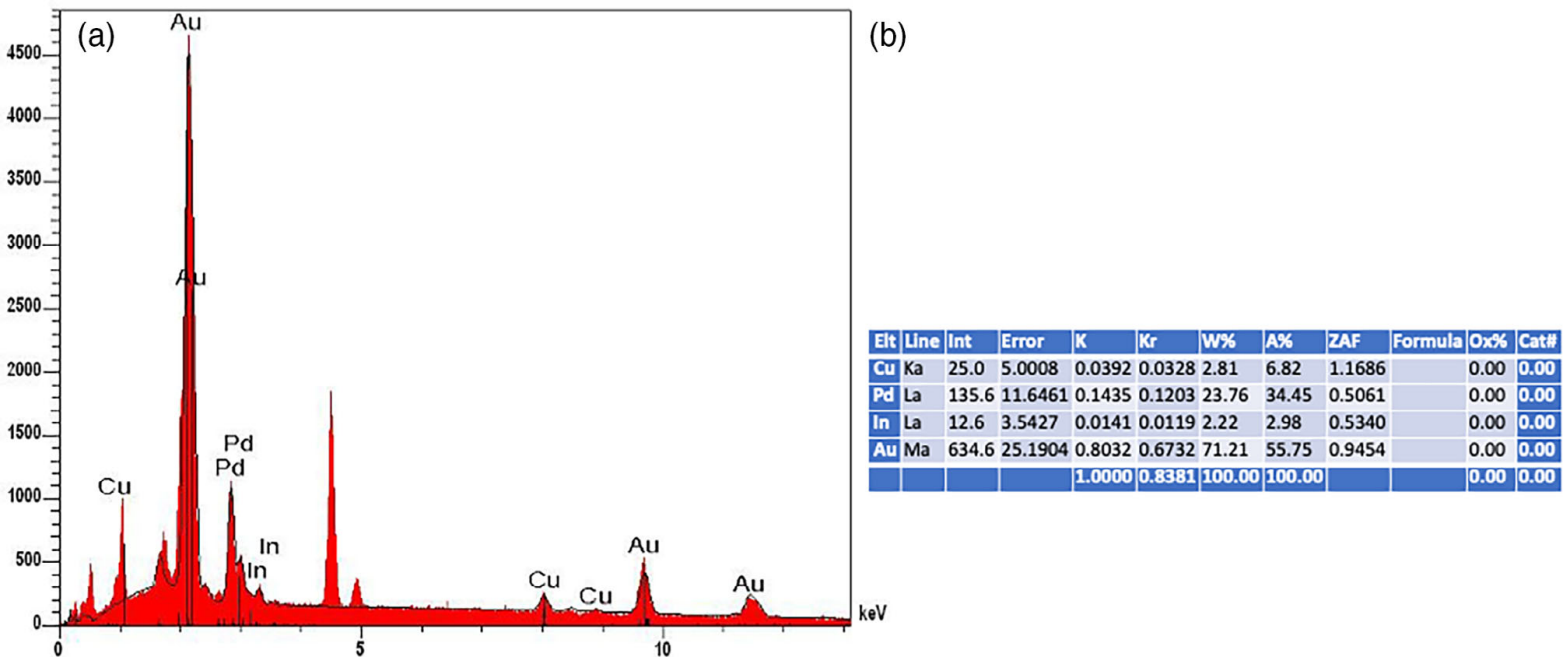
(a and b) Graph shows a gold deposit on the surface of the implant found in scan no. 12 due to wear and tear during the instrumentation of the IS‐TiP inserts, which are entirely gold‐plated

Despite the limitations of the study to simple size, it would reserve further investigation; therefore, our SEM images showed that mechanical instrumentation could cause significant damage to the implant surface (Figure 6).

Microbiological results show that the less aggressive IS‐TiP‐STS‐3E tip has a significant reduction in bacterial load with a standard deviation of 4%, but lower than the Peek EMS tip, which showed a standard deviation of 8%, while the more aggressive tip showed a poor reduction in bacterial load with a standard deviation of 3%. The microbiological results are again in line with the literature; in fact, several studies point out that the use of a metal tip can be effective in removing bacteria from contaminated surfaces (Park et al., 2013; Toma, Behets, Brecx, & Lasserre, 2018).

However, some authors always recommend the combination with an antimicrobial agent to make the reduction of the bacterial load more effective (Polizzi et al., 2020).

The Gold standard of tips used in oral hygiene maintenance therapy is a tip that is not aggressive towards the implant surface but effective in reducing the bacterial load (Palmer & Floyd, 1995).

Therefore, comparing our results obtained from the morphostructural SEM analysis and the microbiological analysis, the IS‐TiP‐STS‐3E tip is the least aggressive even if it did not obtain the best result in terms of bacterial load reduction. To solve this problem, the literature suggests combining the tip with an antimicrobial agent such as chlorhexidine (Calderini et al., 2013).

With the limitations of the study, we can state that the IS‐TiP‐STS‐3E tip used with a microbial agent that increases its bacterial load reduction power, for example, chlorhexidine, could be a combination therapy of choice during the maintenance of the implant as it is not very aggressive and with a sufficient reduction of the bacterial load.

Furthermore, the hygienist plays a fundamental role in the dental team for home motivational education, in monitoring patients with a pre‐ and post‐operative recall program and in promptly intercepting any problems to be reported to the surgeon.

## 5 CONCLUSIONS

Within the limitations of this study, according to the obtained results, the authors recommend the association between the IS‐TiP‐STS‐3E tip^©^ and an antimicrobial agent. Further long‐term in vitro and in vivo studies are needed to confirm these results.

## CLINICAL RELEVANCE

### Scientific rationale for the study

Nonresident bacteria in the oral cavity are responsible for many oral diseases; professional oral hygiene in patients who have implant rehabilitation is critical to minimize the risk of peri‐implantitis and avoid implant failure.

### Principal findings and practical implications

Although the literature is unfavorable to ultrasonic metal tips, our results tell that TiP STS‐3E metal tip associated with a microbial agent not only minimizes the presence of bacterial colonies but surprisingly is not aggressive to the implant surface.

## CONFLICT OF INTEREST

All the authors declare that there are no conflicts of interest regarding the publication of this paper.

## AUTHOR CONTRIBUTIONS

Elisabetta Polizzi and Simone Tomasi conceived idea; Bianca D'orto and Giulia Tetè collected and analyzed the data. Elisabetta Polizzi reviewed the paper and was the supervisor.
